# The IUPHAR/BPS Guide to PHARMACOLOGY in 2024

**DOI:** 10.1093/nar/gkad944

**Published:** 2023-10-28

**Authors:** Simon D Harding, Jane F Armstrong, Elena Faccenda, Christopher Southan, Stephen P H Alexander, Anthony P Davenport, Michael Spedding, Jamie A Davies

**Affiliations:** Centre for Discovery Brain Science, Deanery of Biomedical Sciences, University of Edinburgh, Edinburgh EH8 9XD, UK; Centre for Discovery Brain Science, Deanery of Biomedical Sciences, University of Edinburgh, Edinburgh EH8 9XD, UK; Centre for Discovery Brain Science, Deanery of Biomedical Sciences, University of Edinburgh, Edinburgh EH8 9XD, UK; Centre for Discovery Brain Science, Deanery of Biomedical Sciences, University of Edinburgh, Edinburgh EH8 9XD, UK; School of Life Sciences, University of Nottingham Medical School, Nottingham NG7 2UH, UK; Experimental Medicine and Immunotherapeutics, University of Cambridge, Cambridge CB2 0QQ, UK; Spedding Research Solutions SAS, Le Vésinet 78110, France; Centre for Discovery Brain Science, Deanery of Biomedical Sciences, University of Edinburgh, Edinburgh EH8 9XD, UK

## Abstract

The IUPHAR/BPS Guide to PHARMACOLOGY (GtoPdb; https://www.guidetopharmacology.org) is an open-access, expert-curated, online database that provides succinct overviews and key references for pharmacological targets and their recommended experimental ligands. It includes over 3039 protein targets and 12 163 ligand molecules, including approved drugs, small molecules, peptides and antibodies. Here, we report recent developments to the resource and describe expansion in content over the six database releases made during the last two years. The database update section of this paper focuses on two areas relating to important global health challenges. The first, SARS-CoV-2 COVID-19, remains a major concern and we describe our efforts to expand the database to include a new family of coronavirus proteins. The second area is antimicrobial resistance, for which we have extended our coverage of antibacterials in partnership with AntibioticDB, a collaboration that has continued through support from GARDP. We discuss other areas of curation and also focus on our external links to resources such as PubChem that bring important synergies to the resources.

## Graphical Abstract

**Graphical Abstract:**
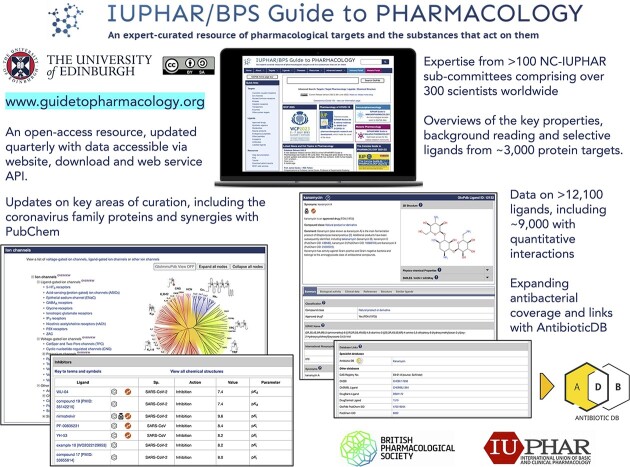

## Introduction

The Guide to PHARMACOLOGY (GtoPdb) was established and developed by the International Union of Basic and Clinical Pharmacology (IUPHAR) and the British Pharmacological Society (BPS). It originated from the IUPHAR-DB, a former resource focused on receptors and channels that was first compiled in 2003 (1–3) and from the BPS ‘Guide to Receptors and Channels’ (4), a compendium of a wider range of targets than those originally covered in IUPHAR-DB. These two resources were merged to form the Guide to PHARMACOLOGY in 2011. The combined resource has continued to expand its coverage of target families and quantitative target-ligand interactions, with guidance and oversight maintained through the Nomenclature and Standards Committee of the International Union of Basic and Clinical Pharmacology (NC-IUPHAR). Overall, throughout the course of its development, GtoPdb has involved input from cumulatively over 1000 scientists from 109 NC-IUPHAR subcommittees (5) and presently the database has around 325 active contributors. GtoPdb is also the source of the Concise Guide to Pharmacology, a biennial publication, which is a tabular extract of the database providing concise overviews of the key properties of nearly 1900 human drug targets with an emphasis on selective pharmacology (6).

The scope of the database expanded in two Wellcome Trust-funded projects to cover the data-supported druggable human genome (7) and immunopharmacology. This later aspect was made accessible to immunologists via the dedicated portal, the IUPHAR Guide to IMMUNOPHARMACOLOGY (GtoImmuPdb; https://www.guidetoimmunopharmacology.org) (8–10). More recently the Guide to MALARIA PHARMACOLOGY (GtoMPdb; https://www.guidetomalariapharmacology.org) (11), was funded as a collaboration between IUPHAR and Medicines for Malaria Venture (MMV; https://www.mmv.org). Our most recent funded collaboration is with AntibioticDB (ADB; https://www.antibioticdb.com) (12), supported by the Global Antibiotic Research and Development Partnership (GARDP; https://gardp.org/) (13,14). Antimicrobial resistance is one of the top threats to global health (https://www.who.int/news-room/fact-sheets/detail/antimicrobial-resistance) and it has been estimated that nearly 1.3 million people die annually as a result of drug-resistant bacteria (15), a figure that exceeds both HIV and breast cancer. The AntibioticDB database benefits early-stage research and development by providing valuable references and starting points for future research and for re-development of discontinued agents. Our collaboration with ADB presents an opportunity to strengthen both resources. One aim is to extend antibacterial compound coverage in GtoPdb and to build reciprocal links between the two resources. Through this partnership we will also develop tools that improve access to antibacterial pharmacology.

Since our last update in 2022 (16) we have made six database releases. In this paper, we describe the key recent curatorial updates for ligand, target and interactions in the database. We discuss specific efforts for both Coronavirus pharmacology, with the expansion of Coronavirus family proteins, and for antibacterial ligands as part of our collaboration with ADB. We also describe some of the ways we aim to ensure GtoPdb remains FAIR-compliant, and how users can access our data. This covers how GtoPdb is closely integrated with other key resources, such as ChEMBL (17,18) and PubChem (19), and we show how this brings important added value for users.

## Guide to PHARMACOLOGY database updates

### Summary of content and curation

Curation and database development is led by the GtoPdb Curation Team (https://www.guidetopharmacology.org/about.jsp#curation) based at The University of Edinburgh, UK. Our specialised and stringent approach to curation involves expertise at all stages and is conducted in collaboration with NC-IUPHAR subcommittees. GtoPdb's strength lies in this discerning, independent and expert-led curation and the prioritisation of data validated from independent sources. A key feature of the GtoPdb is the speed with which new content is released to the live website. We provide four releases each year, which offers our users access to information about new therapeutic strategies in a timely manner. Table 1 shows a summary of targets, ligands and interactions curated in the latest release of GtoPdb (v2023.2, 07 Aug 23). Online database reports compiled to accompany our biannual IUPHAR/BPS combined meetings, provide additional details. The latest of these, at the time of writing, covers November 2022 to April 2023 (https://doi.org/10.5281/zenodo.7915909).

**Table 1.** Guide to PHARMACOLOGY data counts for targets, ligands and interactions from database release 2021.3 (August 23)

**A. Target class content. Human UniProtKB accession counts**	
GPCRs	399 (0)
Nuclear hormone receptors	48 (0)
Catalytic receptors	253 (0)
Ion channels	278 (0)
Transporters	555 (0)
Enzymes	1246 (+31)
Other proteins	229 (+13)
Total number of targets	3039 (+44)
**B. Ligand category counts**	
Synthetic organics	8551 (+958)
Metabolites	509 (-7)
Endogenous peptides	813 (+10)
Other peptides including synthetic peptides	1494 (+73)
Natural products	403 (+69)
Antibodies	354 (+37)
Inorganics	39 (0)
Approved drugs	1918 (+230)
Withdrawn drugs	109 (+21)
Coronavirus	100 (+18)
Antibacterials	471 (+168)
WHO essential list	301 (+19)
Antimalarial	136 (+22)
Ligands with INNs	3311 (+427)
PubChem CIDs	9852
PubChem SIDs	12 163
Total number of ligands	12 163 (+1138)
**C. Interaction counts**	
Human targets with ligand interactions	1947 (+100)
Human targets with quantitative ligand interactions	1695 (+99)
Human targets with approved drug interactions	732 (+58)
Primary targets* with approved drug interactions	347 (+12)
Ligands with target interactions	10 137 (+913)
Ligands with quantitative interactions (approved drugs)	8959 (+798)1107 (+89)
Ligands with clinical use summaries (approved drugs)	3494 (+489)1910 (+226)
Number of binding constants	51 346 (+1515)
References	43 997 (+2956)

*A reduction in the number of metabolites is a consequence of curatorial reassessment.

**Primary target indicates the dominant Molecular Mechanism of Action (MMOA). The table includes a comparison to the figure in the 2022 update (16). Categories are not mutually exclusive, and targets and ligands can fall into more than one.

The changes from the 2021.3 (September 2021) release are shown in parenthesis.

#### Ligands

Since our last update (16), we have added 1142 new ligands to the database. These additions are summarised in Table 2. The table compares total ligand counts from our previous update and indicates where ligands, already curated in GtoPdb, have been updated under the categories in the table. We have added 16 antimalarial compounds and 142 antibacterial compounds. Across all ligand categories a total of 158 new approved drugs were added. Overall, 67% (772) of new ligands have quantitative data on their interactions with their targets.

**Table 2.** Summary of new ligands added to GtoPdb in the 2023.2 database release (August 2023) compared with the 2021.3 release (September 2021)

	New ligands	Updated ligands	Total ligands (2023.2)	Total ligands (2021.3)
Approved drugs	158	72	1918	1688
WHO essential medicines	8	11	301	282
Antibacterials	142	26	471	303
Ligands with quantitative interaction data	772	26	8959	8161
Antimalarials	16	6	136	114
All ligands	1142	-	12 163	11 025

‘New ligands’ column shows count of new ligands for each category; ‘Updated ligands’ shows count of existing ligands, already curated in GtoPdb, now included in the categories.

New ligands are curated from several sources:

- Ligands submitted as part of database updates from our NC-IUPHAR target family subcommittees (e.g. a tranche of around ∼80 were added in the second quarter of 2023 during compilation of the forthcoming 2023/2024 Concise Guide to Pharmacology)
- Carefully monitoring journals with a high density of past entries. Foremost of these is the Journal of Medicinal Chemistry where the inclusion of SMILES for lead compounds is particularly useful for curation. We also monitor social media for relevant new papers.
- New ligands from the proposed INN lists, in conjunction with new target identifications as outlined below. Curators interrogate various sources (PubChem, ChEMBL, patents, pharmaceutical company pipeline resources) to map the INNs to SMILES, company research codes, interaction data and where relevant, clinical potential.
- New ligands with antibacterial activity, curated as part of our collaboration with ADB.
- Inspection of the open Drug Hunter (https://drughunter.com) and Dalriada (https://dalriadatx.com/articles/) monthly lists of novel clinical leads plus their ‘first disclosures’ from sessions at scientific meetings. These are only curated if the *in vitro* activity is openly provenanced (even if patent-only) and we update our records with eventual journal publications.
- New ligands for existing targets extracted from these same resources. Examples include novel chemotypes for kinase inhibitors, ligands with novel or alternate mechanisms of action (exemplified by protein degrader molecules as alternatives to small molecule inhibitors or monoclonal antibodies, and mutant-selective kinase inhibitors as targeted therapies), novel monoclonal antibodies (or antibody fragments) with modified actions (bi/trispecifics, antibody-drug conjugates) and first drug approvals for specific diseases (for example, the anti-C5 monoclonal antibody pozelimab, which was the first therapeutic option to be authorised by the FDA to treat CHAPLE disorder).
- Information regarding the approval status of drugs directly from the FDA (https://www.fda.gov/drugs/new-drugs-fda-cders-new-molecular-entities-and-new-therapeutic-biological-products/novel-drug-approvals-2023), EMA (https://www.ema.europa.eu/en/medicines), other accessible national agencies’ online portals, and from the ‘Drugs’ journal (https://www.springer.com/journal/40265).
- New compounds of interest highlighted by our collaboration with the Probes & Drugs database and the Chemical Probes Portal (for which one of us, CS is a reviewer) (20).
- The BJP instructions for authors specify the linking of ligands and targets to our GtoPdb entries in the eventual on-line HTML and PDF manuscripts. A small number of these turn out to be novel, for which we curate a new entry as part of the author submission process and update with the eventual BJP PMID.

Including selected sources outside peer-reviewed literature, while maintaining our curatorial stringency, allows the GtoPdb to accumulate novel ligand data at a faster pace than could be achieved by relying on published articles alone. In conjunction with our quarterly GtoPdb release schedule, our approach facilitates the rapid dissemination of curated, evidence-based pharmacology content to the wider scientific community, both on the GtoPdb website and via our PubChem updates.

To extract chemistry from papers and patents the curators use open tools such as DECIMER (https://decimer.ai/) (21) to generate SMILES from 2D chemical images and OPSIN (University of Cambridge; https://opsin.ch.cam.ac.uk/) (22) to convert IUPAC names to SMILES. For larger molecules published as defined peptides (including venoms) or approved therapeutic polynucleotides we use Sugar & Splice (NextMove Software; https://www.nextmovesoftware.com) to generate SMILES. All ligand SMILES strings are entered into GtoPdb as the primary chemical structure identifiers and used to map our ligands to PubChem and ChEMBL but we also include InChIs (strings and keys) for expanded interoperability and searching options.

#### Targets

Targets in GtoPdb use a UniProtKB/SwissProt (23) accession as their primary identifier and are organised into hierarchical target families. Table 1A shows the count of targets curated in GtoPdb against different top-level target classes, showing a total of 3039 targets, with a human UniProtKB ID, an increase of 44 since our last update (31 enzymes, 13 other protein targets). A summary of some of the new protein targets that have been curated into the GtoPdb since our last NAR update is shown in Table 3. In addition, nine new target IDs were assigned to bacterial, malarial and coronavirus proteins. These non-human proteins therefore lack the Human UniProtKB identifiers used for the count in Table 1A.

**Table 3.** Summary of 30 new protein targets that have been curated into the GtoPdb since our last NAR update, alongside ligands that have been published as modulators of their activity

GtoPdb ID	Protein name	Class	Ligand	Ligand type*	Proposed use
3203	protein tyrosine phosphatase non-receptor type 11	ENZ	batoprotafib	ssm	oncology (Phase 2)
3199	palmitoyl-protein thioesterase 1	ENZ	ezurpimtrostat	ssm	oncology; viral infection (Phase 2)
3192	coagulation factor III, tissue factor	ENZ	tisotumab vedotin	ADC	oncology (Phase 2)
3239	glutathione peroxidase 4	ENZ	ML162	ssm	oncology
3235	S-phase kinase associated protein 2	ENZ	compound 14i [PMID: 37204466]	ssm	oncology
3234	Cbl proto-oncogene B	ENZ	C7683	ssm	oncology
3228	ubiquitin conjugating enzyme E2 D1	ENZ	EN450	ssm	oncology
3230	DNA polymerase theta	ENZ	RP-6685	ssm	oncology
3224	protein tyrosine phosphatase 4A3	ENZ	JMS-053	ssm	oncology
3215	CTP synthase 1	ENZ	compound 27 [PMID: 36449304]	ssm	oncology
3210	branched chain amino acid transaminase 1	ENZ	BAY-069	ssm	oncology
3209	ubiquitin specific peptidase 8	ENZ	compound 61 [PMID: 36221183]	ssm	oncology
3223	ubiquitin specific peptidase 21	ENZ	BAY-805	ssm	oncology
3207	phosphodiesterase 12	ENZ	compound 1 [PMID: 26055709]	ssm	viral infection
3201	chymotrypsin like elastase 2A	ENZ	elafin	endogenous peptide	microbial infection
3194	HtrA serine peptidase 1	ENZ	galegenimab	mAb	wet AMD/geographic atrophy (Phase 2)
3233	F-box protein 3	ENZ	BC-1215	ssm	inflammatory respiratory disease/injury
3222	hydroxysteroid 17-beta dehydrogenase 13	ENZ	BI-3231	ssm	nonalcoholic steatohepatitis
3211	diacylglycerol O-acyltransferase 2	ENZ	ervogastat	ssm	nonalcoholic steatohepatitis (Phase 2)
3245	ATP citrate lyase	ENZ	NDI-091143	ssm	metabolic disorders; oncology
3205	nicotinamide N-methyltransferase	ENZ	NNMT inhibitor 14 [PMID: 35904556]	ssm	metabolic disorders; oncology
3236	ketohexokinase	ENZ	PF-06835919	ssm	metabolic disorders
3200	selectin E (CD62E)	OP	uproleselan	glycomimetic ssm	oncology (Phase 3); COVID-19 pneumonia (Phase 1/2)
3212	folate receptor alpha	OP	mirvetuximab soravtansine	ADC	oncology (Phase 3)
3195	clusterin	OP	sotevtamab	mAb	oncology (Phase 2)
3240	TEA domain transcription factor 1	OP	GNE-7883	ssm	oncology
3237	axin 2	OP	CW85319	ssm	oncology
3232	BCL3 transcription coactivator	OP	BCL3 inhibitor JS6	ssm	oncology
3213	sequestosome 1	OP	YTK-2205	ssm	proteinopathies
3196	transferrin receptor (CD71)	OP	pabinafusp alfa	fusion protein; iduronate 2-sulfatase enzyme replacement therapy with targeted CNS delivery mediated by fusion with an anti-CD71 Fab fragment	MPS type II (Phase 3)

*Ligand type abbreviations: ADC (antibody-drug conjugate), AMD (age-related macular degeneration), CNS (central nervous system), ENZ (enzyme), mAb (monoclonal antibody), MPS (mucopolysaccharidosis), OP (other protein target), ssm (synthetic small molecule).

The proposed clinical applications are given in the ‘Proposed Use’ column. The majority of the ligands are experimental tools or preclinical leads. Those that are in clinical development have the maximum phase of development shown in brackets.

One of the key objectives of GtoPdb is to provide information on the human targets of approved drugs. When the GtoPdb was established the main target groupings were GPCRs, ion channels, nuclear hormone receptors, catalytic receptors, transporters and enzymes. As new protein targets emerged that did not ‘fit’ into these categories, the ‘Other protein targets’ section was added. The enzymes section has expanded to include kinase and proteases families along with previously unlisted enzymes as tractable targets for specific diseases. Changes have also been made to the enzymes section with expansion to the kinase and protease families, in part reflecting the increase in clinical compounds curated for these important targets (24,25). As a general rule, GtoPdb curators add new protein targets when there is robust evidence of direct pharmacological modulation at therapeutically-relevant concentrations and accompanying *in vivo* data implying potential clinical utility. These new protein targets are extracted from a number of sources:

- Peer reviewed literature, including primary medicinal chemistry journals and clinical and therapeutic journals, are the principal resources for data curation.
- From patents, which includes those suggested to us through our informal collaboration with BindingDB (26).
- The WHO publishes two lists of proposed INNs each year from which new targets are identified via the mechanism of action provided for each compound.

#### Interaction data

**Figure 1.**
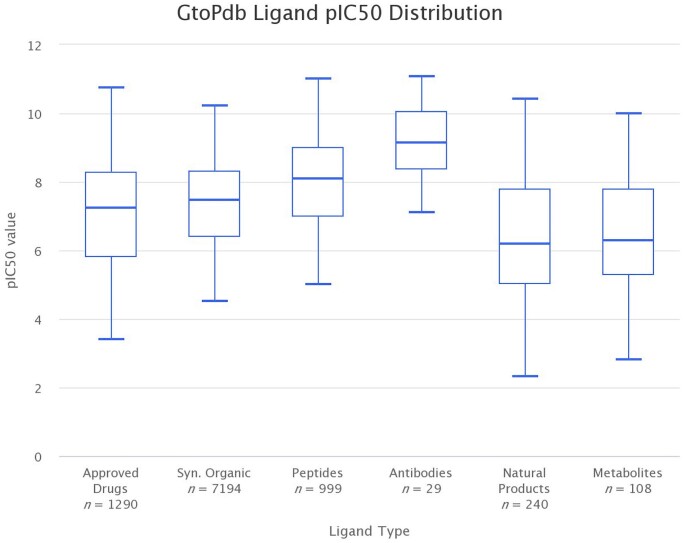
Box plot summary of extracted pIC50 values for ligand types in GtoPdb. The plot shows six data series, each one for a different ligand type. Each series summarises the pIC50 values for interactions involving ligands of that type. The minimum and maximum whiskers are set to be 1.5*IQR (Interquartile range). Charts were prepared using Highcharts JS (https://www.highcharts.com).

**Figure 2.**
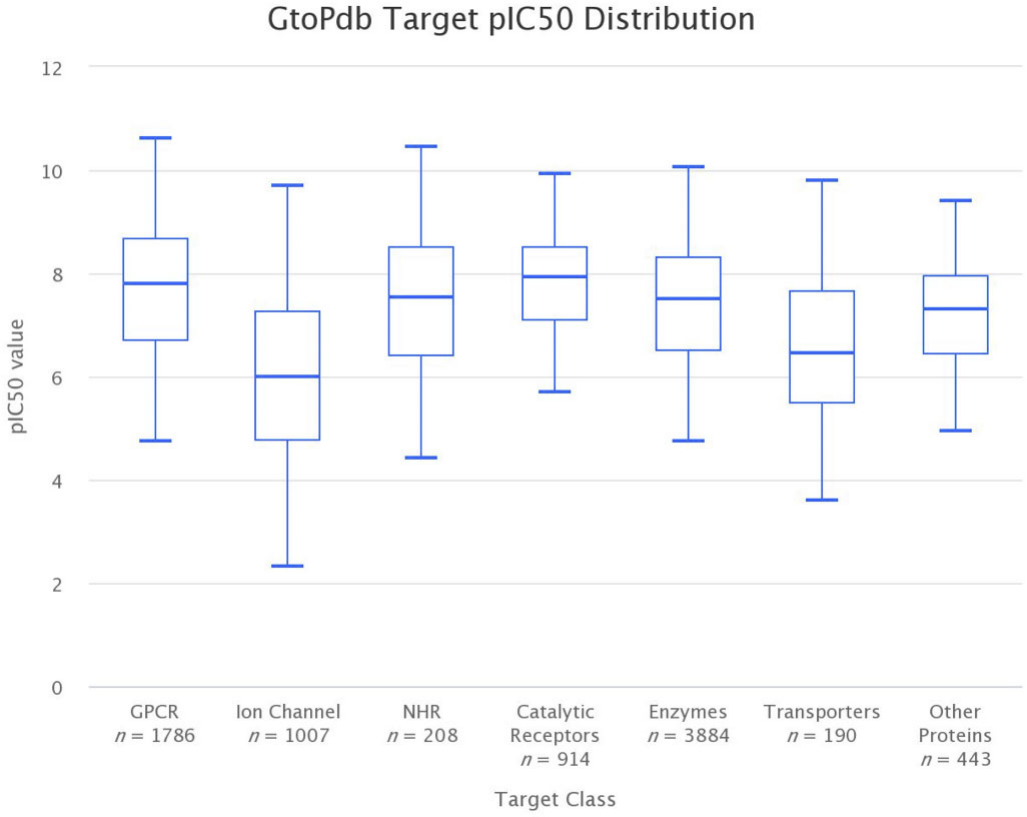
Box plot summary of extracted pIC50 values for target classes in GtoPdb. The plot shows six data series, each one for a different target class. Each series summarises the pIC50 values for interactions involving targets of that class. The minimum and maximum whiskers are set to be 1.5*IQR (Interquartile range). Charts were prepared using Highcharts JS (https://www.highcharts.com).

Curating quantitative interactions is the cornerstone of the data in the GtoPdb. Table 1C shows that there is at least one curated ligand interaction for each of 1947 human protein targets, 1695 of which have quantitative binding data. Restricting this analysis of human targets with quantitative binding data to approved drugs shows 732 interactions, 347 of which are where the protein is the primary target of the drug. Curation concentrates on the most potent and well characterised compounds, typically one from each paper. Each individual ligand-target interaction has a value and a parameter, as recorded from the primary reference. In total there are 22 430 quantitative interaction data points in GtoPdb, covering over 8000 curated ligands. An analysis of interaction data in GtoPdb show ∼70% (15492) are a pIC50 or pKi value. Figure 1 uses Highcharts JS (https://www.highcharts.com) to draw box plots that summarise the pIC50 values of GtoPdb interaction data involving different ligand types. It shows that the median pIC50 is highest for antibodies (9.15), which also has the highest interquartile range (8.32–10.05) and shows the least variance. The median pIC50 for peptides is 8.11, synthetic organic ligands is 7.48, approved drugs is 7.23, metabolites is 6.30 and natural products is 6.20. The spread of values is broadest within the natural products group. Figure 2 summarises pIC50 values for interactions involving different protein target classes. The median pIC50 interquartile range is highest for GPCR (7.81) and Catalytic Receptors (7.92) and lowest for Ion Channels (6.00) which also has the broadest spread. The pIC50 distributions indicate the spread of activity values curated in GtoPdb under different target classes and ligand classifications. These can be useful, for example, to compare approved drug activity distributions to compounds under investigation. Identifying compounds below the approved drug distribution range may indicate those less likely to prove effective and provide a rationale for not progressing further.

### Coronavirus pharmacology

The GtoPdb Coronavirus Information page (https://www.guidetopharmacology.org/GRAC/CoronavirusForward) was created in early 2020, as a space for IUPHAR to host reliable information relating to the scientific and clinical advances being made towards SARS-CoV-2 antivirals and drug-based treatments for COVID-19 (16). The urgency for such developments has waned, largely as a result of the success of vaccination programmes, and the identification of effective drug therapies for severe COVID-19 thorough randomised controlled trials such as the international RECOVERY clinical trial (https://www.recoverytrial.net/). The GtoPdb curators do however continue to monitor the medicinal chemistry and clinical literature, review patents and explore other sources (e.g. the open COVID drug discovery project ‘COVID Moonshot’ https://postera.ai/moonshot/; the WHO INN Proposed Lists of COVID-specific agents https://www.who.int/teams/health-product-and-policy-standards/inn/inn-lists) to add new ligands and therapeutic targets when the data meet our curation criteria.

The most intensively pursued coronavirus target proteins, as judged by the number of published inhibitors, remain the 3C-like (main) protease (Mpro) and the RNA-dependent RNA polymerase (RdRP), although some groups have developed tool inhibitors for the virus’ second protease, papain-like protease (PLpro) (27–29). GtoPdb has now curated 130 Mpro inhibitors, including the approved drugs nirmatrelvir (the Mpro inhibitor in Paxlovid®) and ensitrelvir. These all have interaction data from papers and/or patents. A curated set of X-ray crystal structures that show inhibitors in complex with Mpro has also been included. Representative Mpro PROTAC degrader molecules were extracted from patents (US11530195B1, US11518759B1) as a novel approach to providing anti-SARS-CoV-2 activity. The GtoPdb dataset contains structures for five RdRP inhibitors, only one of which, remdesivir, is approved for clinical use by the US FDA and the EU EMA. We maintain an active collaboration for CoV-2 target curation with BindingDB who primarily curate SAR from patents (e.g. for Mpro inhibitors) (26). This means we can add patent number pointers to our Mpro inhibitors where BindingDB has curated the complete SAR set. For example we included the reference to WO2021250648 in our nirmatrelvir entry which BindingDB have located as example 13 from US11351149 with 95 curated Ki values https://www.bindingdb.org/jsp/dbsearch/PrimarySearch_ki.jsp?tag=entry&entryid=10640.

Initial research objectives were focussed towards developing antiviral drugs, and therapies to treat the myriad symptoms of COVID-19. The underlying biological mechanisms and individual factors that confer risk and susceptibility to developing severe COVID-19 have been intensely investigated; much is understood, but there remain many unknowns. In view of the predicted prevalence of long-term debilitating illness post-SARS-CoV-2 infections (long COVID) our monitoring for possible curation encompasses the identification of the mechanisms involved in long COVID (30,31), and how these might be ameliorated by pharmacological interventions. At the time of writing diagnostic and treatment options for long COVID are limited, clinical studies involving drug interventions are few, and funding for long COVID research is restricted (32).

### Antibiotic DB/GARDP collaboration

GtoPdb has been collaborating with Antibiotic DB (ADB; https://www.antibioticdb.com) (12) since 2019. The interaction improved the coverage of antibacterial compounds in GtoPdb and provided chemistry and pharmacology for the antibacterial compounds curated within ADB. The initial phase saw a curatorial effort to map compounds in ADB with ligands in GtoPdb, typically done by a combination of name, PubChem CID and SMILES identifier mapping. We also added any antibacterials in ADB that were not already in GtoPdb and met our threshold for inclusion. Each new antibacterial ligand entry in GtoPdb was manually curated to include external database links, mechanism of action information, clinical approval status and bioactivity data relating to the antibacterial potency of the ligand against pathogens. As indicated in Table 1 there are 471 ligands now tagged in GtoPdb as ‘antibacterial’ as a result of this curatorial effort, with 168 ligands being added since our last update (Table 2). The collaboration has also brought added value by setting up direct links between GtoPdb ligand summary pages and ADB compound records. The collaboration is continuing with plans to develop a bespoke portal for users to access data most relevant to antibacterial pharmacology. The curation and development team in Edinburgh is also working with ADB to rebuild the front-end of their website.

## Guide to PHARMACOLOGY website updates

### FAIR and machine-readable downloads

Provision of data in simple reusable formats is essential in helping to ensure GtoPDB is FAIR-compliant (Findable, Accessible, Interoperable, Reusable) (33). The FAIR Principles emphasise improving the ability of machines to automatically find and use data, in addition to supporting its reuse by individuals. GtoPdb ensures that it is FAIR-compliant, so that its data and resources can be of maximum benefit in supporting and improving knowledge for pharmacological research and beyond, and is a named FAIR-sharing resource (https://fairsharing.org/FAIRsharing.f1dv0) (34).

The GtoPdb download page (https://www.guideopharmacology.org/download.jsp) provides comma-separated and tab-delimited files (compatible with multiple spreadsheet packages) of GtoPdb targets, ligand, interactions and more. Across the site, we have also added additional buttons to allow users to download specific data sets. On average, data files are downloaded from GtoPdb ∼360 times a month. We also make available our PostgreSQL dump file of the entire database. We have continued to populate the endogenous/natural ligand pairing file. This file contains all ligands and ligand subunits considered endogenous and the protein target they interact with. The file includes ligand and target UniProtKB and Ensembl IDs, and also includes indications of the rank potency of the ligand and any more detailed curatorial comments. A separate file has also been added that includes quantitative interaction data for the endogenous ligand-target pairings. Downloads of this nature provide useful views of GtoPdb data, highlighted by their use by GPCRdb (35,36) to support the development of a new GPCR ligand resource (37). Providing the different machine-readable options above makes the outputs of our stringent data curation available for Artificial Intelligence (AI) exploitation (38).

### Chemical search

We have updated our chemical structure search (https://www.guidetopharmacology.org/GRAC/chemSearch.jsp#structureSearch) to now use the Chemistry Development Kit (CDK) (https://cdk.github.io/) to power similarity searches. CDK is a collection of free and open-source modular Java libraries for processing chemical information (39).

## Guide to PHARMACOLOGY usage and connectivity

### Site usage and impact

GtoPdb is an open-access and free resource that aims to provide accurate information on the basic science underlying drug action to support research and education. It continues to be very well accessed by users, which we can track via Google Analytics. In the 12 months to 29th March 2023 the site has seen a monthly average of ∼52425 sessions from ∼36960 users and an average of 146694 page views per month. Although access to GtoPdb is dominated by the USA, China, UK and India (∼56% of users) access comes from across the globe. In the 12 months to 29th March 2023, a total of 69 different countries recorded over 500 sessions. Our two most recent NAR database reports from 2020 and 2022 (11,16), according to European PubMed Central impact statistics, have acquired a combined 121 citations and an Altmetric score of 23.

### External links

GtoPdb has had a collaboration with MMV, in the development of the IUPHAR/BPS Guide to Malaria Pharmacology, for the malaria research community to provide open and optimised access to published data on malaria pharmacology (16,40). We are also, as earlier described, currently collaborating with GARDP and AntibioticDB. Beyond these more formal collaborations, GtoPdb maintains numerous connections with other resources, many of which are reciprocal in terms of cross-pointing web links. Notably, and discussed in more detail later, are our links with PubChem (19) where we submit substances after each database release. We also have strong connections with ChEMBL (17,18), with ChEMBL data used to supplement our pharmacology search and ligand activity charts. In terms of reciprocal connectivity ChEMBL chemistry records include links to GtoPdb ligands via the EBI UniChem resource (https://www.ebi.ac.uk/unichem) (41). We link all targets to UniProtKB (23) as the primary identifier (except for viral polyproteins where we provide a TrEMBL target-specific excised sequence actually used in the screening assays). We submit a cross-reference file to UniProtKB at each database release and outlinks are provided as one of their six Chemistry database link sets (https://www.uniprot.org/database?query=*facets=category_exact%3AChemistry+databases), so it is possible when searching UniProtKB to filter by ‘GuidetoPharmacology’. We have a long-standing collaboration with HGNC (42) on shared nomenclature interests and our curatorial adherence to mapping HGNC approved human gene symbols and names to NC-IUPHAR nomenclature.

In addition, we have built links between more specialist resources such as the previously mentioned GPCRdb, Reactome (43) for metabolites plus pathways and RESOLUTE for solute carriers (44).

GtoPdb submits data to NCBI via their link-out utility, which indexes our PMIDs (https://pubmed.ncbi.nlm.nih.gov/?term=loprovguidpharm[SB]) with additional links for Genes, Nucleotides and Proteins. We also submit to Europe PMC (https://europepmc.org/) through their external links service (https://europepmc.org/LabsLink). Using this service, links are added from Europe PMC articles to related GtoPdb target and ligand data - where the article is a reference to a curated pharmacological interaction in GtoPdb. A full list of the 8198 Europe PMC articles with GtoPdb data links (at the time of writing) can be retrieved from https://europepmc.org/search?query=%28LABS_PUBS%3A%221969%22%29

Wherever we have cross-links to other resources we maintain contacts with the teams concerned and do our best to ensure these are regularly refreshed. This includes providing ftp files to be picked up but there can be synchronisation delays. For example, our target cross-references update in UniProt every two months which may cover multiple GtoPdb releases.

### PubChem

**Figure 3.**
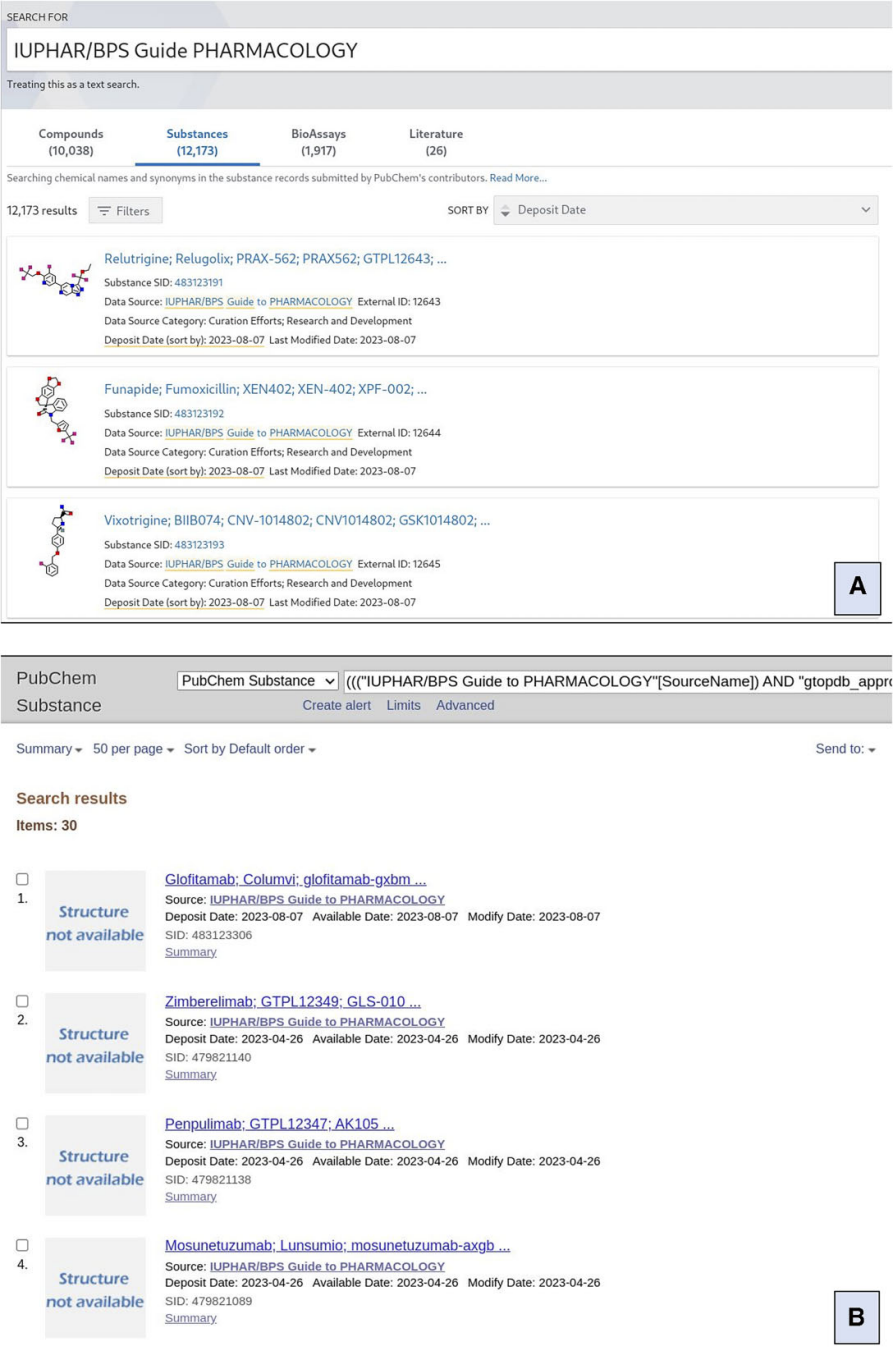
Guide to PHARMACOLOGY data in PubChem. Panel A shows the result of a main search on PubChem for ‘IUPHAR/BPS Guide to PHARMACOLOGY’. Panel B shows the result of searching PubChem substances for Guide to PHARMACOLOGY compounds, made available since 2020, tagged with our approved drug and antibody categories. Full query used is (((‘IUPHAR/BPS Guide to PHARMACOLOGY’[SourceName]) AND ‘gtopdb_approved’[Comment]) AND ‘gtopdb_antibody’[Comment]) AND (‘2020/01/01″[AvailableDate] : ‘3000’[AvailableDate]).

The powerful utilities arising from GtoPdb ligand submissions and consequent data integration into PubChem (19) and other NCBI resources (45), have already been described (8). PubChem Substances (SIDs) are community-submitted structures with information about the molecule. PubChem merges unique chemical structures from different SIDs as PubChem Compounds (CIDs). Using the PubChem Substance interface, the newest GtoPdb entries from the 2023.2 release have increased the Substance Identifier (SID) count to 12173, as shown in Figure 3A via searching PubChem with the source term "IUPHAR/BPS Guide to PHARMACOLOGY''. This query also indicates that there is a PubChem Compound (CID) count of 10 038. The difference between these two totals is because some ligand SIDs are too large to form CIDs because they exceed the chemical structure specification upper limit of ∼70 amino acid or nucleotide polymeric units (our largest CID is agatolimod, an oligodeoxynucleotide TLR9 agonist with a MW of 7707). Thus, these 2135 SID-only ligands include antibodies, other protein ligands, plus larger peptides and polynucleotides without CIDs and unusual chemical modifications not easily convertible to SMILES. The following statistics can be discerned from the 10038 CIDs with at least one of our SIDs. For 91 of these GtoPdb is the single-submitter out of the 116 million CIDs in PubChem. This uniqueness has several origins of which the predominant one is our high release frequency (e.g. 66 of these have been added since April 2023). Another reason is the isomeric complexities associated with ∼25 higher MW peptide and nucleotide entries going back to before 2023, some of which may have CIDs from other submitters with different isomeric assignments. We have been extending our ligand tagging to enable users to retrieve and compare sets of particular interest as described in this blog post https://cdsouthan.blogspot.com/2022/11/guide-to-pharmacology-selectable.html).

**Figure 4.**
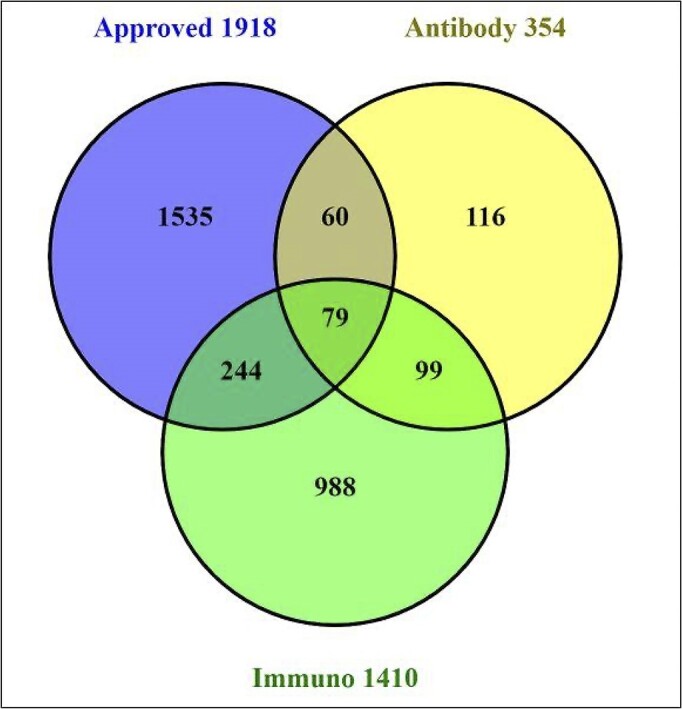
Venn diagram showing ligand PubChem SID intersections between approved drugs, antibodies and records from the Guide to Immunopharmacology. Diagram prepared using Venny (https://bioinfogp.cnb.csic.es/tools/venny/).

Selections of five different tags are shown in Table 4. The approved drugs include 186 SID-only entries. Of these, 113 are antibodies, with the difference being large peptides or polynucleotides. Similarly, the immunopharmacology SIDs include 173 antibodies but the antimalarials just two, meplazumab and the CIS43 antibody. Queries of our content can be combined as Boolean operations (as can any PubChem interface queries). The result shown in Figure 3B shows the result of the combined query ‘approved drug’, ‘antibody’ and ‘available after 1st Jan 2020’. Figure 4 shows the Venn intersections of three queries, in this case ‘approved drug’, ‘antibody’ and ’immunopharmacology’. The web tool Venny (https://bioinfogp.cnb.csic.es/tools/venny/) is particularly useful for isolating and analysing segment-specific lists. The CID entries for GtoPdb can be browsed, interrogated, filtered and intersected (i.e. finding entries in common between other CID collections) with 100s of other data sources. PubChem has also added co-occurrence recommendations (46).

**Table 4.** Counts of GtoPdb tagged ligands in PubChem (August 2023), using selected substance queries (column 2)

Ligand type	Query*	SID Count	CID count
Approved drugs	gtopdb_approved [comment]	1918	1698
Immunopharmacology	gtopdb_immuno [comment]	1410	983
Antimalaria	gtopdb_malaria [comment]	136	134
Antibacterial	gtopdb_antibacterial [comment]	469	468
Antibody	gtopdb_antibody [comment]	354	0
Natural Products	gtopdb_natural_product [comment]	311	301

*Example query format for approved drugs ‘‘IUPHAR/BPS Guide to PHARMACOLOGY’[SourceName] AND ‘gtopdb_approved’[comment]’.

CID numbers are retrieved via the ‘Find related data’ > ‘Database’ > ‘PubChem Compound’ > ‘PubChem Same Compound’ > CID count and display (i.e. a SID > CID conversion).

### Comparison with other resources

**Figure 5.**
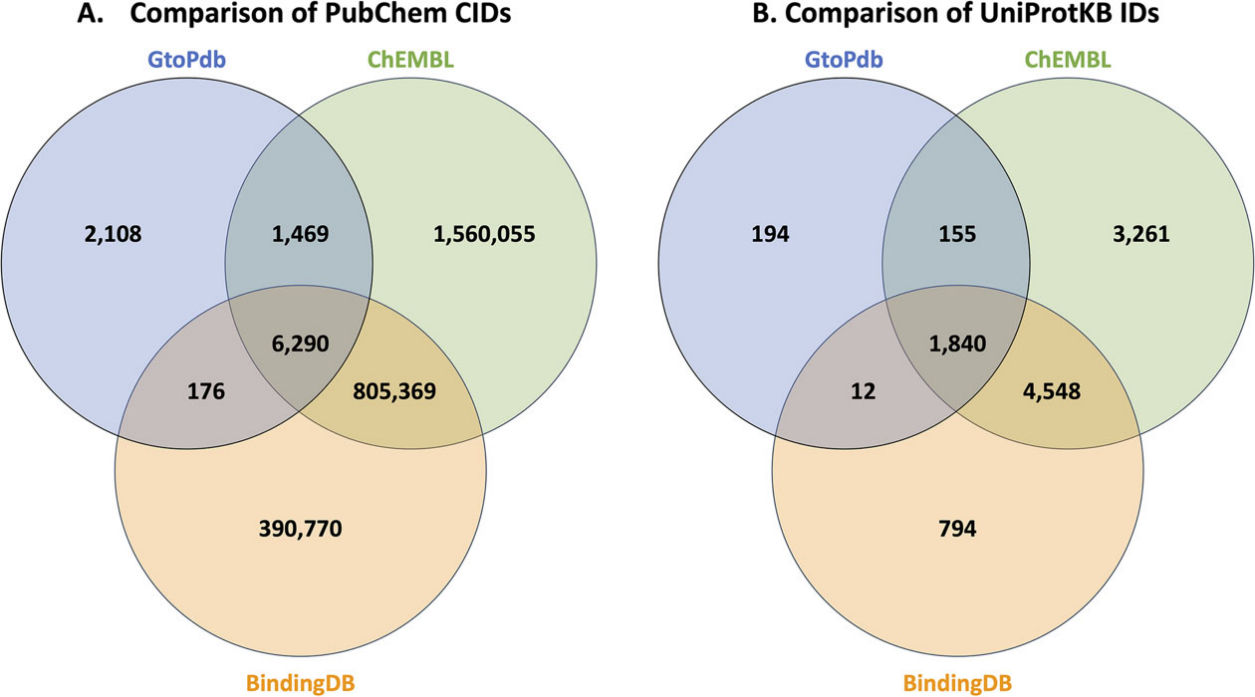
Venn diagrams showing the comparison of PubChem CIDs (**A**) and UniProtKB identifiers (**B**) between GtoPdb, ChEMBL and BindingDB. CID counts are taken using the advanced PubChem Compound search (https://www.ncbi.nlm.nih.gov/pccompound), specifying source name in the query (i.e. ‘IUPHAR/BPS Guide to PHARMACOLOGY’[SourceName] NOT ‘ChEMBL’[SourceName]). UniProtKB counts are taken from the UniProtKB advanced search, filtering on Cross-Reference > Chemistry Database (i.e. https://www.uniprot.org/uniprotkb?query=%28database%3Aguidetopharmacology%29+NOT+%28database%3Achembl%29). The update frequency of these cross-references may be variable depending on the sources.

It is useful to compare GtoPdb with ChEMBL (17) and BindingDB in terms of complementarity for users. While there is variation in data models and query functionality of the individual resources, content comparison is possible as all three submit to PubChem and have target cross-references in UniProtKB. Figure 5 shows the comparison of PubChem CIDs and UniProtKB between GtoPdb, ChEMBL and BindingDB. Around 23% of GtoPdb compounds do not overlap with ChEMBL. ChEMBL extracts all assay data, including ADMET determinations, from a paper whereas GtoPdb usually extracts just the lead compound but will also curate reported secondary target activity. In the comparison with BindingDB, 36% of GtoPdb compounds do not overlap. BindingDB’s uniqueness is mainly their patent curation; it also has an arrangement with ChEMBL from which it subsumes just the individual protein target-mapped data. GtoPdb target overlap with both ChEMBL and BindingDB is extensive, GtoPdb has 206 not in ChEMBL and 349 not in BindingDB.

## Future directions

Enabled by funding from GARDP we will establish a new portal, similar in approach to the Guide to Malaria Pharmacology, to direct users to antimicrobial/antibacterial content in the database. For example, we are looking at categorising antibacterial data based around antibiotic classification and to provide links with the GARDP REVIVE encyclopaedia (https://revive.gardp.org/resources/encyclopaedia/). We will continue to measure the impact and value of the database for the global scientific community. A significant recent development has been in the rapid increase in pre-prints that have not yet undergone peer review, particularly those reporting results for SARS-CoV-2 and COVID-19. Our objective will be to maintain a high-standard of curation by tracking the inclusion of pre-prints in the database and whether a subsequent reviewed paper is published or retracted. Opportunities to support and/or sponsor the Guide to Pharmacology are available, if interested please get in touch at enquiries@guidetopharmacology.org.

## Data access

GtoPdb, GtoImmuPdb and GtoMPdb are available online at https://www.guidetopharmacology.org, https://www.guidetoimmunopharmacology.org and https://www.guidetomalariapharmacology.org, respectively. All three resources are licensed under the Open Data Commons Open Database License (ODbL) (https://www.opendatacommons.org/licenses/odbl/) and the contents are licensed under the Creative Commons Attribution ShareAlike 4.0 International (CC BY-SA 4.0, https://creativecommons.org/licenses/by-sa/4.0/). Advice on linking to us and for accessing and downloading data are provided here: https://www.guidetopharmacology.org/linking.jsp. GtoPdb aims to make 3 to 4 public database releases per year; the data summaries and statistics reported in this paper are from release 2023.2 (July/August 2023). Our downloads page (available from https://www.guidetopharmacology.org/downloads.jsp) provides a dump file of the full PostgreSQL database, in addition to several specific download files for targets, ligands, interactions, peptides, endogenous/natural ligands, and approved drugs with primary targets, ligand ID mapping, ligand SDF file and RDF flat files. Our REST web services are available at https://www.guidetopharmacology.org/webServices.jsp and provide computational access to data in JavaScript Object Notation (JSON) format.

## Citing the resource

This publication replaces all previous papers for citing this resource. Citation advice for specific target pages appears on the website. Please refer to our resources on first mention by full correct name (IUPHAR/BPS Guide to PHARMACOLOGY, IUPHAR Guide to IMMUNOPHARMACOLOGY, IUPHAR/MMV Guide to MALARIA PHARMACOLOGY), including the capitalization. For subsequent abbreviation, please use GtoPdb, GtoImmuPdb and GtoMPdb, specifying the release version number (this can be found on our About page - https://www.guidetopharmacology.org/about.jsp#content).
